# Molecular mechanisms of the anchang group prescription in treating radiation enteritis: network pharmacology analysis and experimental evidence

**DOI:** 10.3389/fphar.2025.1524925

**Published:** 2025-04-08

**Authors:** Wei Liang, Bo Li, Yuehong Sun, Dapeng Jia, Tingting Hu, Rujing Huang, Zhilong Liu, Huan Yang, Baocai Chen, Xiaoming Yin, Xinying He, Yunchuan Sun

**Affiliations:** ^1^ Hebei Province Integrated Traditional Chinese and Western Medicine 3D Printing Technology Innovation Center, Department of Oncology, Hebei Province Cangzhou Hospital of Integrated Traditional and Western Medicine, Cangzhou, Hebei, China; ^2^ Department of Traditional Chinese Medicine, Graduate School of North China University of Science and Technology, Tangshan, Hebei, China; ^3^ Department of Traditional Chinese Medicine, Cangzhou Medical College, Cangzhou, Hebei, China

**Keywords:** anchang group prescription, radiation enteritis, network pharmacology, PI3K/akt pathway, intestinal barrier

## Abstract

**Background:**

The “Anchang” Group Prescription (ACZF), based on the traditional Bai Tou Weng Decoction and Si Jun Zi Decoction, has demonstrated clinical efficacy in alleviating symptoms of radiation enteritis (RE). Nevertheless, the precise active components and their underlying molecular mechanisms in ACZF’s effect on RE require further elucidation. This investigation seeks to delineate the active components and explore the molecular mechanisms by which ACZF mitigates RE, utilizing both network pharmacology and experimental approaches to provide a solid theoretical base for subsequent research and clinical applications.

**Methods:**

Utilizing network pharmacology, this research constructed a comprehensive “drug-active ingredient-target gene-disease” model leveraging resources such as TCMSP, SwissTargetPrediction, GeneCard, and OMIM. Cytoscape 3.8.2 along with the STRING database were instrumental in developing a protein-protein interaction (PPI) network for the identification of pivotal targets. Functional enrichment analyses, including Gene Ontology (GO) and Kyoto Encyclopedia of Genes and Genomes (KEGG) pathways, were conducted via the DAVID database. Experimentally, a mouse model of RE was induced by X-ray exposure to assess the physiological and pathological responses. Parameters measured included body weight, survival rate, incidences of diarrhea, and hematochezia; histological assessments involved hematoxylin and eosin (H&E) and Masson’s trichrome staining to examine morphological alterations and collagen deposition in colonic tissues. Levels of cytokines such as interleukin-1β (IL-1β), IL-6, IL-10, and tumor necrosis factor-α (TNF-α) were quantified using enzyme-linked immunosorbent assays (ELISA). Additionally, immunohistochemistry (IHC) and Western blotting (WB) were employed to evaluate the expression of tight junction proteins zonula occludens-1 (ZO-1) and claudin-1, as well as proteins linked to the PI3K/AKT pathway.

**Results:**

Key bioactive constituents of ACZF in treating RE include quercetin, kaempferol, isorhamnetin, and luteolin, with core target proteins such as SRC, STAT3, AKT, HSP90AA1, and EGFR. Involved signaling pathways include PI3K/AKT, RAP1, and MAPK. *In vivo* results revealed that mice treated with ACZF showed enhanced survival, increased body weight, and extended colon lengths compared to controls. Although Masson staining showed no significant differences, H&E staining indicated that radiation-induced mucosal damage, including extensive ulcer formation, inflammatory cell infiltration, crypt structure destruction, and epithelial layer injury, could all be ameliorated by ACZF. Notable reductions were observed in TNF-α, IL-1β, and IL-6 levels, while IL-10 levels saw a significant rise. There was also a marked increase in the expression of ZO-1 and claudin-1. WB analyses demonstrated the activation of the PI3K/AKT pathway in RE, which was significantly curtailed by ACZF, lowering phosphorylation levels within the colonic tissues. Concurrent administration of the PI3K activator YS-49 with ACZF reversed the inhibitory effects on the PI3K/AKT pathway and mitigated impacts on epithelial TJ protein expression and inflammatory cytokine levels, highlighting the critical role of the PI3K/AKT pathway in mediating ACZF’s therapeutic effects in RE.

**Conclusion:**

ACZF alleviates RE by inhibiting PI3K/AKT activation, reducing inflammation, and preserving intestinal mucosal integrity.

## 1 Introduction

RE frequently manifests in patients receiving radiotherapy for abdominal and pelvic tumors, presenting typically with symptoms such as acute or chronic diarrhea, rectal bleeding, and tenesmus. These manifestations significantly impair the quality of life of cancer patients. Additionally, RE restricts the increase in radiation dosages necessary for effective treatment of rectal and other abdominal malignancies (Ilias et al., 2023). Present treatments for RE primarily comprise probiotics, analgesics, anti-inflammatory medications, and antibiotics (Qilin et al., 2023; Lu et al., 2023). While these approaches are efficacious in alleviating symptoms, their prolonged efficacy is limited.

Traditional Chinese Medicine (TCM) classifies radiation exposure as ‘fire-heat toxic evil,’ attributing the primary pathogenesis of RE to the build-up of heat toxins that damage the intestinal tract. TCM therapeutic strategies for RE emphasize clearing heat, detoxifying, cooling the blood to cease bleeding, and eliminating dampness while harmonizing blood and qi (Zihong et al., 2023). Derived from traditional Bai Tou Weng Decoction and Si Jun Zi Decoction, the ACZF encapsulates these principles. Its properties are comparable to those of antipyretic and anti-inflammatory drugs, promoting tissue regeneration. Clinically, ACZF significantly mitigates symptoms in RE patients and lowers the rate of recurrence (He et al., 2015). Preliminary studies have verified that ACZF also fortifies the intestinal mucosal barrier and diminishes inflammatory indicators in rats afflicted with RE (Sun et al., 2019). However, the active compounds and specific molecular mechanisms of ACZF in RE have not been fully delineated and require extensive investigation. This research aims to delineate the active components and clarify the molecular mechanisms of ACZF in treating RE via network pharmacology and experimental verification, providing a fundamental basis for subsequent research and clinical practice.

## 2 Materials and methods

### 2.1 Collection of active components and targets of ACZF

Active constituents of ACZF were obtained from the TCM Systems Pharmacology (TCMSP) database (https://tcmspw.com/tcmsp.php), mandating an oral bioavailability (OB) ≥ 30% and drug likeness (DL) ≥ 0.18. The Swiss Targets Prediction platform (http://www.swisstargetprediction.ch/) was engaged to identify the targets of these components, specifically targeting “*Homo sapiens*.” Targets with a likelihood ≥0.11 were selected from these predictions. Should the effective components possess fewer than 15 targets meeting this probability, the top 15 targets by probability were chosen.

### 2.2 Prediction and screening of targets related to RE

Targets linked to RE were retrieved from the GeneCards (https://www.genecards.org/) and OMIM (https://www.omim.org/) databases, using “ Radiation enteritis” or “radiation enteropathy” as search keywords and specifying “*H. sapiens*” as the species filter.

### 2.3 Construction and analysis of drug-compound-target-disease network

An online Venn diagram tool (http://bioinformatics.Psb.ugent.be/webtools/Venn/) was used to determine the intersection genes between genes related to RE and possible targets of ACZF. The “drug-compound-target-disease” network was created using Cytoscape 3.8.2 software. Nodes in the network stand for drugs and targets, while lines indicate the relationships between them. The network’s topological properties were analyzed using the Network Analyzer plugin, calculating the degree value of each node. The higher the degree value was, the more important active ingredients and target genes were.

### 2.4 Construction and analysis of PPI network

The STRING database was queried for intersection targets with the species “*H. sapiens*” and a confidence level of ≥0.9 set. To build the interaction network, these results were imported into Cytoscape 3.8.2. In order to identify important targets, the Centiscape 2.2 plugin was used to measure the degree, betweenness, and proximity of every node (Zheng-Lan et al., 2023).

### 2.5 Enrichment analysis

Using the DAVID database (https://david.ncifcrf.gov/), GO and KEGG pathway enrichment analyses were performed to investigate possible molecular pathways by which ACZF improves RE. The GO analysis applied a cutoff of p < 0.05, with the top 10 biological processes below this threshold visualized in a bar chart. Correspondingly, the KEGG pathway analysis adhered to a p < 0.05 significance threshold, with the top 30 pathways under this threshold illustrated in a bubble chart by the bioinformatics platform (https://www.bioinformatics.com.cn/).

### 2.6 Drugs and main reagents

ACZF, composed of Sheng Diyu (Sanguisorba officinalis L) (30 g), Baitouweng (Pulsatilla chinensis) (15 g), Huanglian (Coptis chinensis Franch.) (5 g), Baizhu (Atractylodes macrocephala) (15 g), Fuling (Poria cocos (Schw.) Wolf.) (15 g), Xianhecao (Agrimonia pilosa Ledeb.) (30 g), Muxiang (Aucklandia lappa Decne.) (9 g), Chao Baishao (Paeonia lactiflora Pall) (12 g), and Gancao (Glycyrrhiza uralensis Fisch.) (6 g), was sourced from Cangzhou Hospital of Integrated Traditional Chinese and Western of Hebei Province and accredited by a pharmacologist, Professor Baofen Li. More details are shown in Supplementary Table S1. The ACZF formula was soaked in distilled water for 30 min, decocted, and filtered twice. The filtrate was evaporated and concentrated into a decoction containing 4.2 g/mL crude drug and stored at 4°C (Supplementary Figure S1) (Gang, 1998). Masson’s trichrome staining kit was acquired from Solarbio (Batch No. G1340), and ELISA kits for TNF-α,IL-1β,IL-6, and IL-10 were provided by Shanghai Jianglai Biological Technology Co., Ltd. Antibodies for PI3K (No. 10131698) and p-PI3K (No. 10129672) were purchased from Abmart, while those for Akt (No. 10017913), p-Akt (No. 10020148), ZO-1 (No. 66452-1-Ig), and Claudin-1 (No. 13050-1-AP) came from Wuhan Proteintech Co., Ltd. The PI3K activator YS-49 (No. HY-15477) was obtained from MedChem Express.

### 2.7 Quality control of ACZF

Based on another study we are submitting, the analysis of ACZF was conducted using Ultra-high-performance liquid chromatography-tandem mass spectrometry (UPLC-MS/MS). The metabolites were quantified using multiple reaction monitoring (MRM) mode. Analysis results are available in the Supplementary Material (Supplementary Figure S2; Supplementary Table S2).

### 2.8 Establishment of radiation-induced colitis mouse model

Healthy male C57BL/6 mice, aged 6–8 weeks and weighing 18–22 g, were procured from Henan Sike Best Biotechnology Co., Ltd. (SCXK (Yu) 2020-0005). These mice were housed in the Specific Pathogen-Free (SPF) facility at the Institute of Radiological Medicine, Chinese Academy of Medical Sciences. This study was approved by the Ethics Committee of the Cangzhou Hospital of Integrated Traditional Chinese and Western of Hebei Province (Ethical Approval Number: 2020018).

Following a week of acclimatization, the mice were randomly segregated into control, model, ACZF-treated, and ACZF + YS-49 groups. All groups, except the control, underwent X-ray exposure to induce RE. The mice received anesthesia with pentobarbital, were positioned on the operating board, and irradiated over the entire abdomen with 6 MV-X-rays. The irradiation area covered from the xiphoid process to the pubic symphysis, with an area of 3.0 cm × 3.0 cm, while areas not targeted were shielded with lead plates. The irradiation was performed from 100 cm, at 6 Gy/min for 2.5 min, totaling 15 Gy (Dai and Yang, 2023). Following radiation exposure, the ACZF group was administered 0.2 mL of the aforementioned decoction daily for a week. In the ACZF + YS-49 group, this regimen was supplemented with a daily 5 mg/kg intraperitoneal injection of YS-49. Control and model groups received an equivalent volume of saline. Monitoring was conducted daily, noting body weight, survival rates, fecal morphology and color for signs of diarrhea or hematochezia, and the Disease Activity Index (DAI) was maintained (Zhang et al., 2024). The mice were euthanized by cervical dislocation when the treatment was over. After that, their colons were measured, collected, and divided lengthwise; half of them were preserved in formalin, and the other half were stored at −80°C.

### 2.9 Histopathological evaluation

In accordance with predetermined procedures, the colon was fixed in 10% neutral buffered formalin over the night, embedded in paraffin, sectioned at 4 μm, and stained with H&E. The severity of colonic lesions was assessed by examining the extent of inflammatory cell infiltration, mucosal damage, and structural disruptions.

### 2.10 Masson staining of colon in mice

Following the same steps as for stained with H&E, the colon tissue was processed and sectioned. Afterwards, Masson’s trichrome was applied to the sections in accordance with established procedures. A light microscope was used to analyze the collagen fibers within the colon tissues, and their abundance was estimated using ImageJ software.

### 2.11 Assay of cytokine production in colonic mucosa

Levels of IL-1β, IL-6, IL-10, and TNF-α in the colonic mucosa were quantified using ELISA kits in accordance with the manufacturer’s guidelines.

### 2.12 Immunohistochemistry (IHC) analysis

Colonic tissues were fixed in 10% buffered neutral formalin, dehydrated, and paraffin embedded. Sections (5-μm-thick) were deparaffinised and rehydrated, processed by microwave antigen retrieval, and incubated overnight with primary antibodies against ZO-1 (1:500) and claudin-1 (1:500) at 4 °C. Horseradish peroxidase (HRP)-conjugated secondary antibodies (1:2000) were then applied and incubated at room temperature for 25 min. Visualization was achieved using DAB chromogen, followed by hematoxylin counterstaining. The sections were dehydrated, mounted, and finally observed under an optical microscope. Image analysis was conducted using ImageJ software.

### 2.13 WB analysis

Total protein was extracted from colon tissues using RIPA lysate and then quantified using the BCA method. After separating by 10% SDS-PAGE, proteins were then transferred to PVDF membranes. This was followed by a 2-h room temperature blocking with 5% nonfat dry milk. After an overnight incubation at 4°C with primary antibodies against ZO-1 (1:1000), claudin-1 (1:1000), PI3K (1:1000), AKT (1:1000), p-PI3K (1:1000), p-AKT (1:1000), and GAPDH (1:5000), secondary antibodies were incubated and visualized using Enhanced Chemiluminescence (catalog no. P0018; Beyotime Institute of Biotechnology; Cat #ab276131; Abcam, Proteintech, 13409-1-AP; GeneTex, GTX108613). Quantity-One software version 4.6.2 (Bio-Rad Laboratories, Inc., Hercules, CA, USA) was used for densitometry analysis.

### 2.14 Statistical analysis

Data are expressed as mean ± standard deviation (SD). Statistical analysis involved one-way ANOVA for multi-group comparisons and Tukey’s multiple comparison tests for pairwise comparisons. A p-value <0.05 was considered indicative of statistical significance.

## 3 Results

### 3.1 Construction of drug-component-target-disease network

**FIGURE 1:**
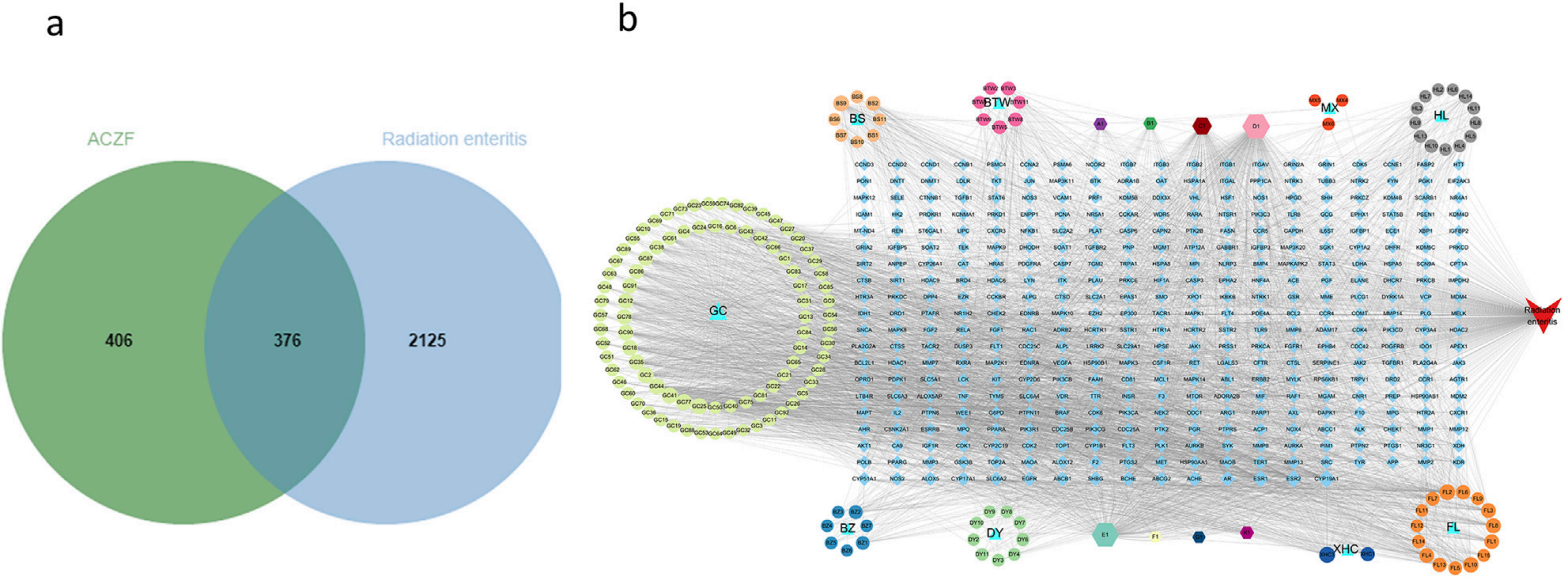
Drug-component-target-disease network of ACZF for RE treatment. **(a)** Venn diagram displays there are 376 common targets of ACZF for RE treatment. **(b)** The ‘Drug-Compound-Target-Disease’ network showcases the interaction between ACZF’s components, RE targets, and disease, underlining the formulation’s multi-target therapeutic potential.

Active components for ACZF were screened from TCMSP database. A total of 159 components and 2124 targets were acquired, following duplicate removal, 782 unique gene targets remained. After merging and eliminating the duplicate targets, there were 2501 targets closely related to RE obtained from GeneCards and OMIM database. By using online Venn diagram tool, 376 intersection targets were obtained (Figures 1a). The drug, ingredients, targets and disease were imported into Cytoscape 3.8.2 to construct the drug-component-target-disease network diagram (Figures 1b), which included 545 nodes and 3,716 edges (Figures 1b). Network analysis indicated extensive interactions among chemical components, reflecting ACZF’s complex mechanism involving multiple pathways and targets. Notably, compounds like Quercetin, Kaempferol, Isorhamnetin, and Luteolin had prominent Degree values, highlighting their pivotal roles in ACZF’s efficacy against RE.

### 3.2 Construction of the PPI network and screening of core target proteins

**FIGURE 2:**
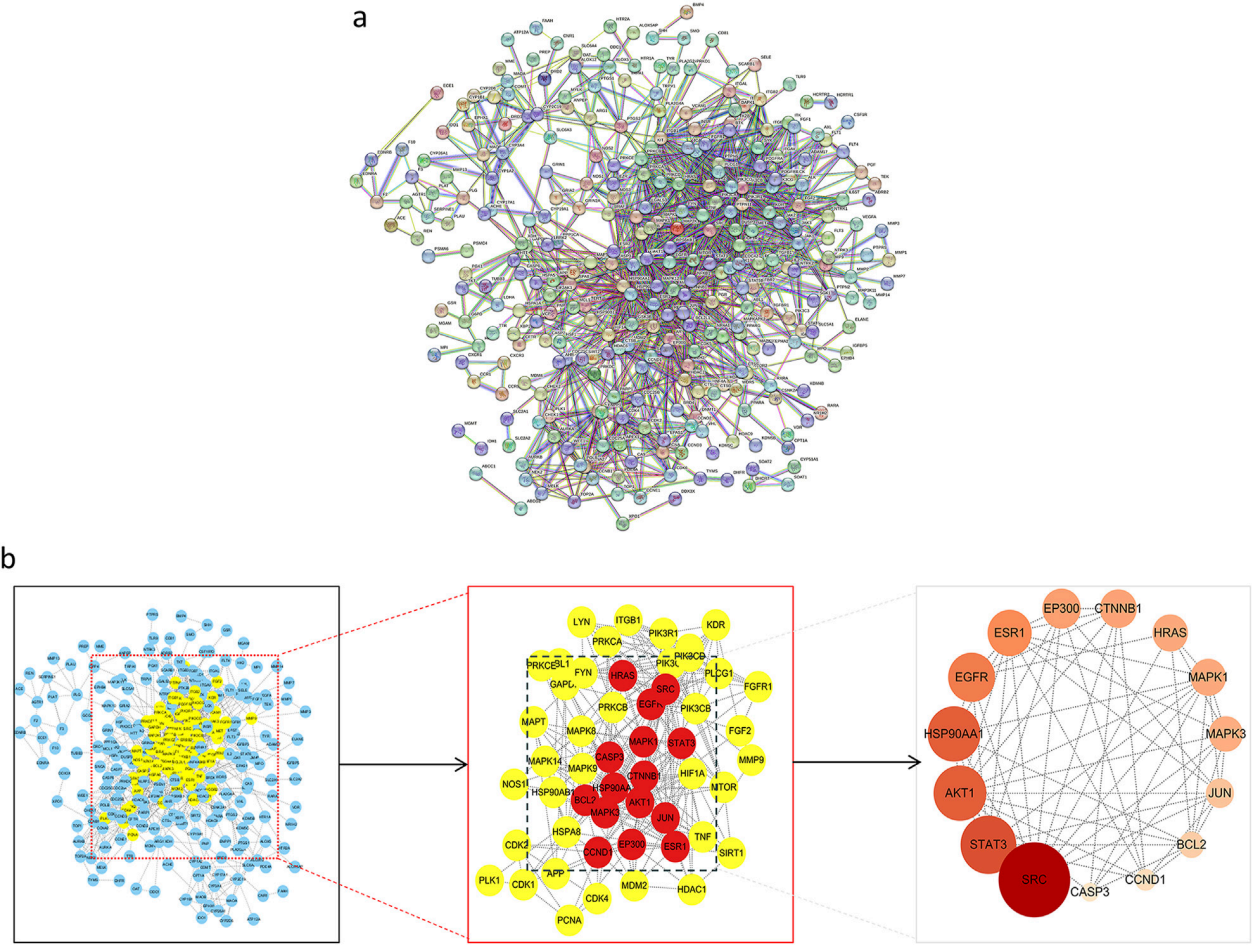
Construction of PPI network and screening of core target of ACZF for RE treatment. **(a)** 376 common target genes were added to the PPI network complex using the STRING database; **(b)** Core target selection.

Target proteins were uploaded to the STRING database to establish a PPI network (Figures 2a), consisting of 301 nodes and 1,382 edges, with an average degree of 9.18. Using the Centiscape 2.2 plugin for topological analysis, 52 candidate targets with a degree ≥9.18 were initially identified. Further refinement based on a degree ≥11.15, betweenness ≥50.58, and closeness ≥0.01 isolated 15 core genes: SRC, STAT3, AKT1, HSP90AA1, EGFR, ESR1, EP300, CYNNB1, HRAS, MARK1, MARK3, JUN, BCL2, CCND1, and CASP3 (Figures 2b).

### 3.3 GO and KEGG analysis

**FIGURE 3:**
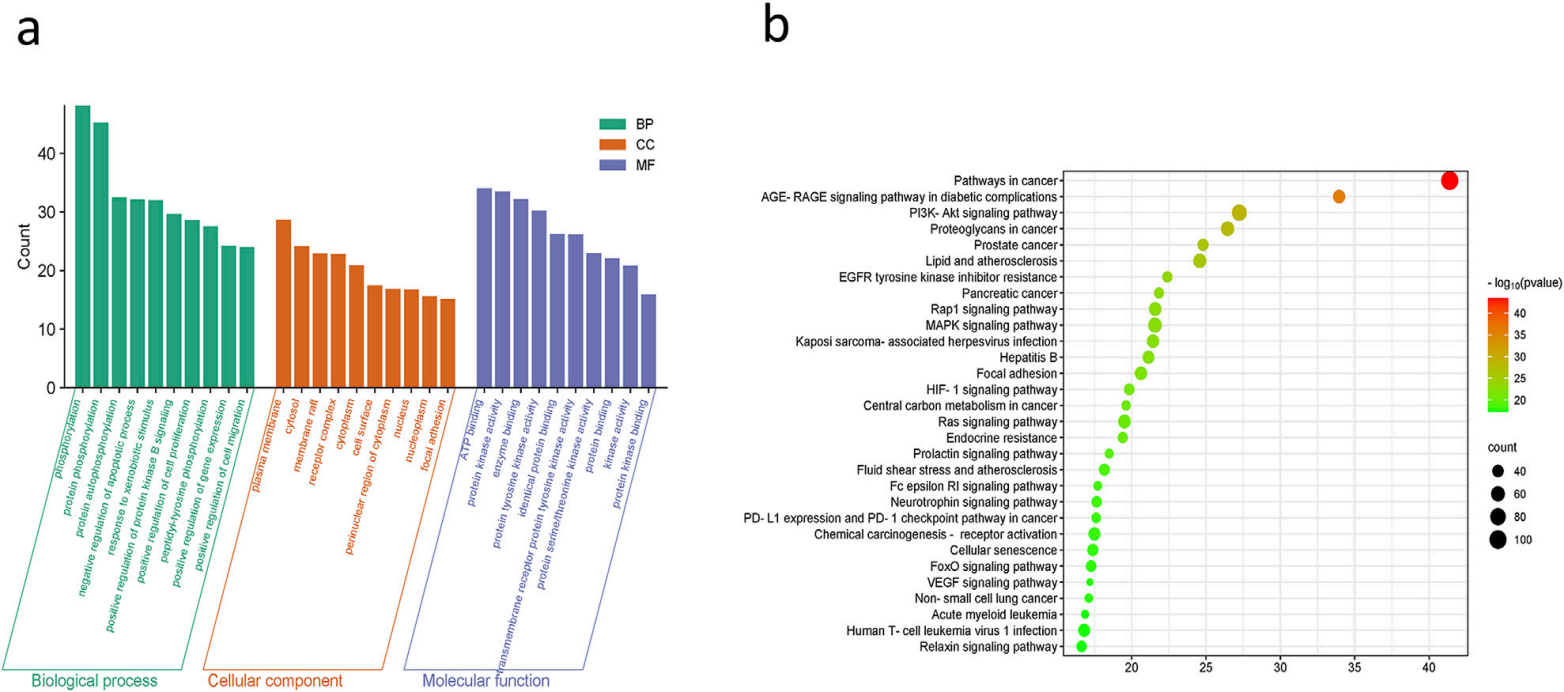
GO and KEGG analysis. **(a)** GO functional analysis and **(b)** enrichment analysis of KEGG pathways.

Based on the results of the DAVID database study, the molecular mechanisms by which ACZF may alleviate RE are mostly involved in the negative control of protein phosphorylation, apoptosis, and cell migration. Protein binding, identical protein binding, and ATP binding were the foci of molecular functions (MF), whereas membrane rafts, receptor complexes, cytoplasm, and the nucleus were the primary cellular components (CC) (Figures 3a). The primary related pathways were concentrated in the AGE-RAGE signaling pathway, PI3K/AKT pathway, RAP1 pathway, MAPK pathway, and FOXO signaling pathway (Figures 3b).

### 3.4 ACZF alleviates radiation-induced colitis in mice

**FIGURE 4:**
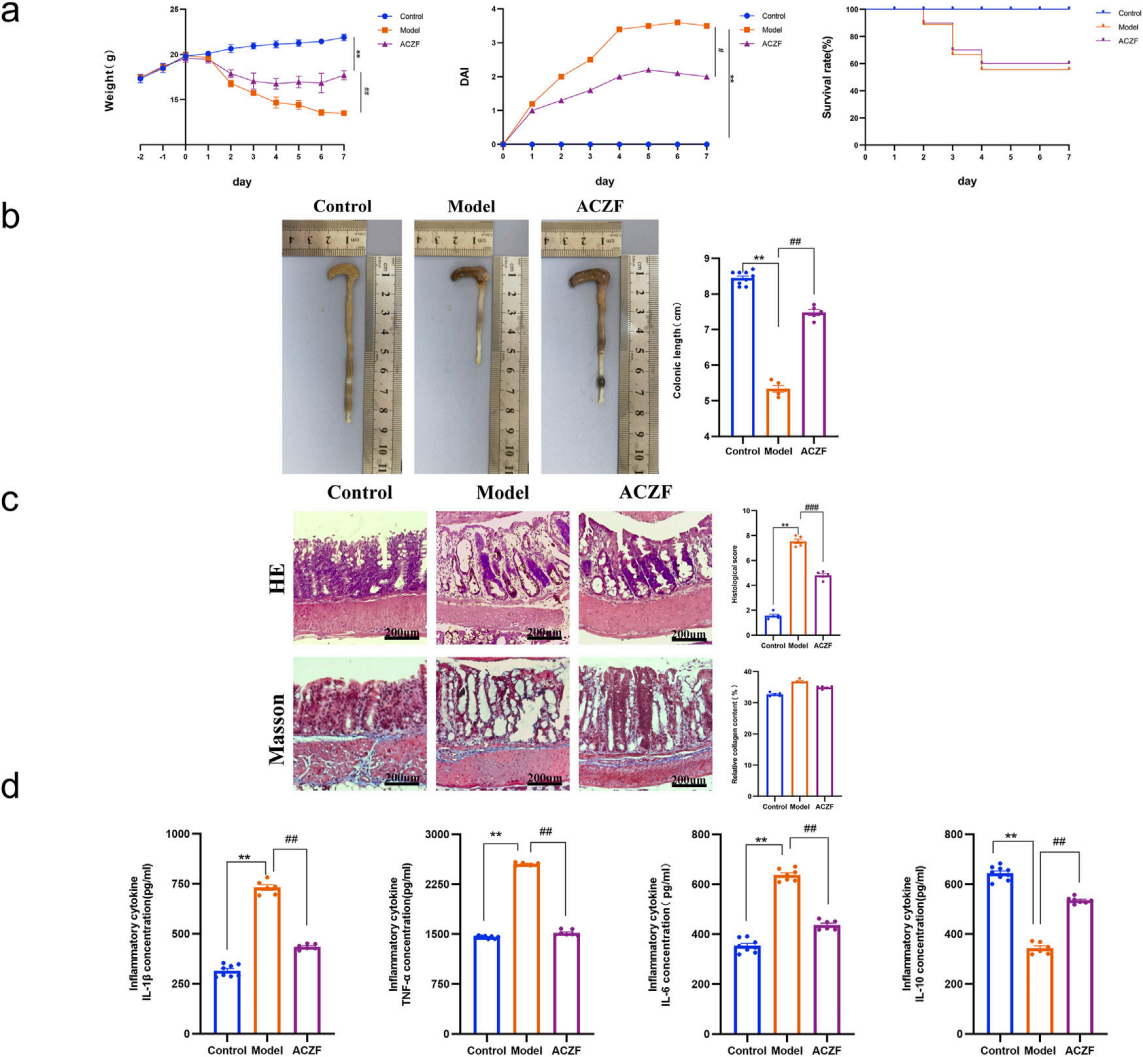
ACZF alleviates the symptoms and colonic inflammation in radiation-induced colitis. **(a)** Daily changes in body weight, disease activity index (DAI) and survival rate in different groups; **(b)** Length of the colon; **(c)** H&E staining and Masson staining of the colon tissue. Scale bar = 200 μm; **(d)** Colonic cytokine levels of TNF-α, IL-1β, IL-6, and IL-10. Data represent means ± SD (n = 8-10). **p < 0.01 vs. Control group; #p < 0.05 vs. Model group; ##p < 0.01 vs. Model group.

Radiation exposure significantly elevated DAI scores, confirming the effective induction of radiation colitis. In contrast, ACZF treatment markedly improved pathological conditions and reduced DAI scores, enhancing the survival rates of mice with RE and demonstrating its therapeutic efficacy (Figures 4a). Measurements of colon lengths, vital for assessing colon inflammation, showed recovery in ACZF-treated mice relative to those with radiation-induced colitis (Figures 4b). H&E staining evaluated morphological and histopathological changes, indicating that radiation caused substantial mucosal ulceration, inflammatory cell infiltration, crypt damage, and epithelial destruction, all significantly alleviated by ACZF. However, based on Masson staining, no significant differences in smooth muscle fibers were observed among the control group, model group, and ACZF-treated group. (Figures 4c). ELISA assessments confirmed that ACZF modulated the inflammatory cytokine profile, reducing pro-inflammatory cytokines (TNF-α, IL-1β, IL-6) and increasing anti-inflammatory cytokine IL-10 levels (Figures 4d). These results further validate ACZF’s capacity to mitigate radiation-induced colitis in mice.

### 3.5 ACZF restores the intestinal barrier function in colitic mice

**FIGURE 5:**
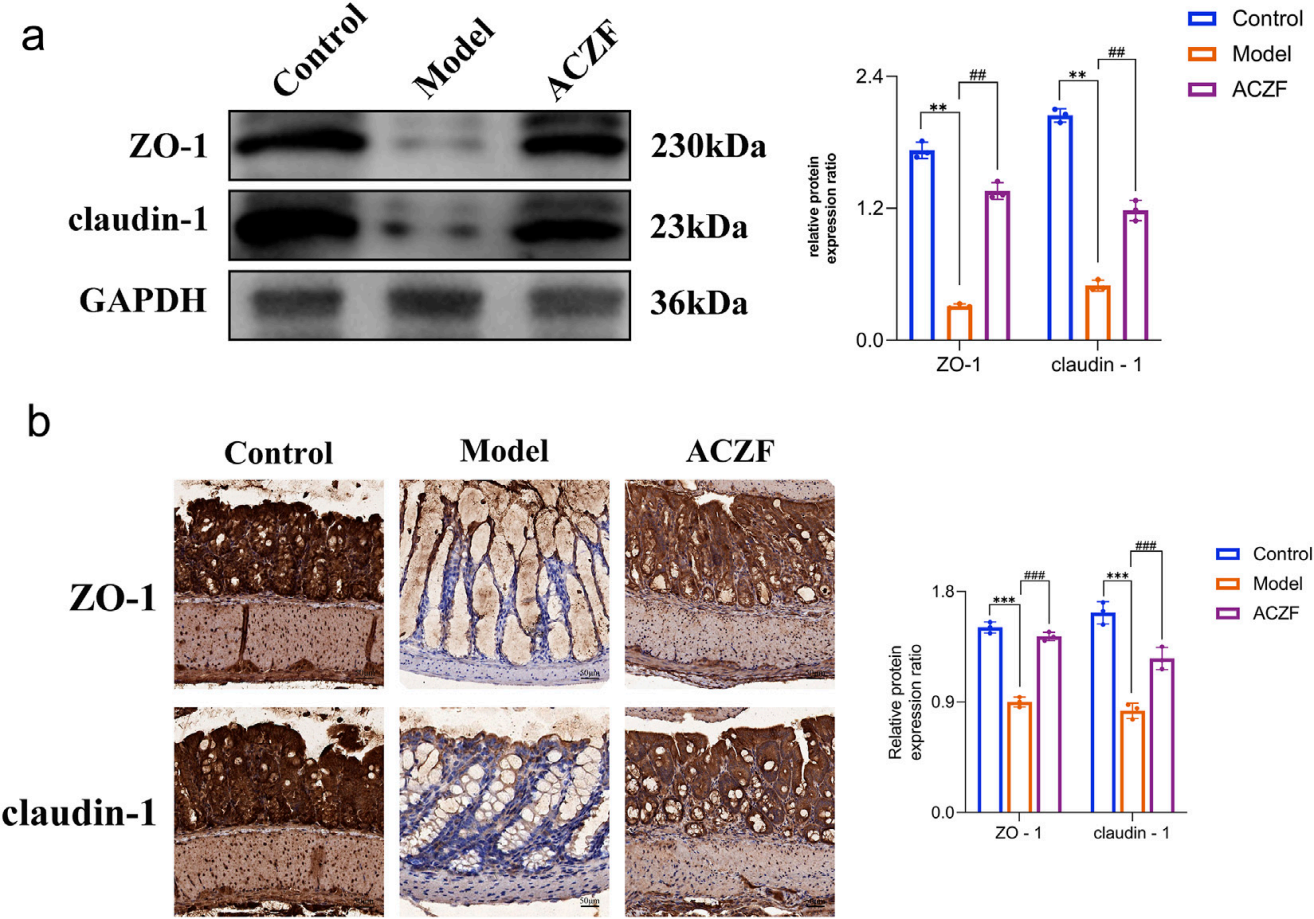
ACZF improves the intestinal barrier function in radiation-induced colitis mice. Western blot analysis **(a)** and immunohistochemical analysis **(b)** of ZO-1 and claudin-1 expression in colon tissue. Data represent means ± SD (n = 4). **p < 0.01 vs. Control group; ***p < 0.001 vs. Control group; ##p < 0.01 vs. Model group; ###p < 0.001 vs. Model group.

To assess the impact of ACZF on epithelial barrier integrity, we examined the expression levels of tight junction (TJ) proteins, specifically ZO-1 and Occludin-1. As shown by the Western blot results, the expression of ZO-1 and Claudin-1 in the colon of RE model mice was significantly reduced compared to the Control group, indicating impaired intestinal barrier function (Figures 5a). Remarkably, the administration of ACZF effectively reversed this condition. Similar results were obtained using immunohistochemistry (Figures 5b). These results indicated that ACZF could effectively restore intestinal barrier function in mice with radiation enteritis.

### 3.6 ACZF improves radiation colitis via pathways involving PI3K/AKT signaling

**FIGURE 6:**
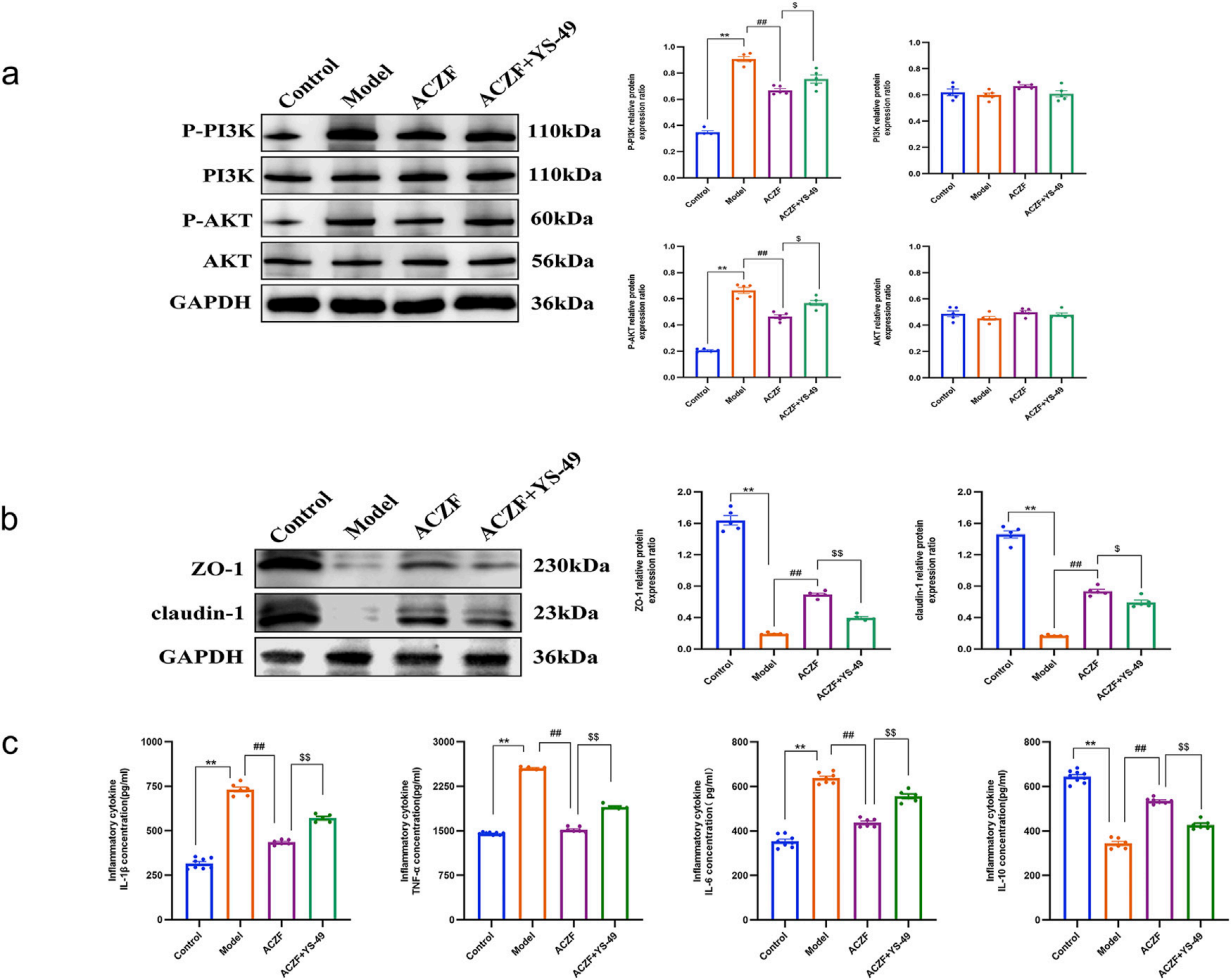
ACZF improves colitis via pathways involving PI3K/AKT signaling. **(a)** Representative immunoblots and the relative expression levels of AKT, p-AKT, PI3K, and p-PI3K in colon tissues from different treatment groups; **(b)** ZO-1and claudin-1 expression in the colon tissues of the colitis mice was detected by Western blot; **(c)** Colonic cytokine levels of TNF-α, IL-1β, IL-6, and IL-10. Data represent means ± SD (n = 4). **p < 0.01 vs. Control group; ##p < 0.01 vs. Model group; $ p < 0.05 vs. ACZF group; $$ p < 0.01 vs. ACZF group.

Initial network pharmacology analysis suggested a possible interaction of ACZF with the PI3K/AKT pathway. To determine if ACZF influences intestinal barrier function via AKT and to evaluate its impact on AKT activation, we conducted a WB analysis. The results indicated that the levels of p-PI3K and p-AKT in the colon mucosal tissues of the model group were markedly elevated, implying that radiation may have activated the PI3K/AKT signaling pathway. Treatment with ACZF markedly reduced this activation (Figures 6a). Additional tests involving simultaneous treatment with ACZF and the PI3K activator YS-49 in a radiation colitis model demonstrated that ACZF’s suppression of the PI3K/AKT pathway, along with its impact on epithelial TJ protein expression and inflammatory marker levels in the colon mucosa, could be reversed (Figures 6b, c), confirming that ACZF ameliorates colitis by inhibiting the PI3K/AKT pathway.

## 4 Discussion

RE commonly occurs as a complication of abdominal and pelvic radiotherapy, manifesting symptoms such as diarrhea, tenesmus, and hematochezia that significantly impair patient quality of life. In some cases, these effects are severe enough to cause patients to interrupt their treatment, negatively impacting radiotherapy outcomes. Research suggests that RE may originate from apoptosis of intestinal mucosal stem cells, irregular inflammatory factor expression, increased intestinal epithelial permeability, dysbiosis, and endothelial damage (Yangyang et al., 2021; Jinjia et al., 2022). The progression of RE involves multiple pathways, including NF-κB, PI3K/AKT, MAPK, and TLR (Lu et al., 2023; Jinsheng et al., 2024; Xiaoyu et al., 2023).

TCM conceptualizes radiation as a “fire toxin” that, when combined with the underlying malignancy, disrupts the body’s Qi, blood, and fluids, leading to organ dysfunction. This condition leads to obstructed Qi mechanisms, accumulation of phlegm and dampness, blockage of meridians, and blood stasis. Such disruptions, exacerbated by radiation, directly affect the gastrointestinal tract, presenting as internal heat toxin, dampness stagnation, and disharmony between Qi and blood, which are fundamental in the pathogenesis of RE. The therapeutic strategy emphasizes clearing heat and detoxifying, cooling the blood to halt bleeding, eliminating dampness, and regulating blood and qi. In ACZF, Sheng Dihuang and Baitouweng primarily clear heat and detoxify; Baizhu and Fuling bolster the spleen, eradicate dampness, and mitigate diarrhea; Huanglian expels heat and dampness from the spleen, stomach, and large intestine; Mu Xiang fortifies the spleen, regulates Qi, and eases stagnation. Chao Bai Shao alleviates acute pain and enriches the blood, while Xian He Cao acts to constrict and halt bleeding, serving supportive roles. Gancao synergizes the effects of all herbs, thereby enhancing spleen strength, stimulating blood circulation, resolving stasis, stopping bleeding, and fostering muscle regeneration.

This study utilized network pharmacology to identify the active components, pivotal targets, and essential pathways of ACZF in the treatment of RE, clarifying possible mechanisms of action. Among the 159 active components identified, notable examples included Quercetin, Kaempferol, and Luteolin. Quercetin, recognized for its antioxidant properties, reduces the release of pro-inflammatory mediators and the expression of inflammatory proteins, thereby lessening inflammation in the intestinal epithelium (Elham et al., 2023; Wei et al., 2023). Kaempferol, a flavonoid, has proven effective in curbing the overproduction of inflammatory mediators in endothelial cells stimulated by lipopolysaccharides (Mohan and Dipankar, 2024). Luteolin impedes the PI3K/AKT signaling pathway, reduces HIF-1α expression, and curtails microvascular formation, thereby mitigating inflammation (Mingtao et al., 2024). Analysis of the PPI network pinpointed potential targets including SRC, STAT3, and AKT1. GO and KEGG pathway analyses indicated that ACZF regulates RE through various signaling pathways, particularly the cancer pathway, AGE-RAGE pathway, and PI3K/AKT pathway. Recent studies highlight the crucial involvement of the PI3K/AKT signaling pathway in the development of colitis, influencing oxidative stress, inflammatory processes, autophagy, and apoptosis (Jin-Ting et al., 2018; Xiaobin et al., 2024). Furthermore, a variety of research has shown that certain TCM practices can modulate the PI3K/AKT pathway, leading to the inhibition of inflammatory mediators and cytokines, which in turn affects colitis (Minghe et al., 2024; Haoren et al., 2021).

Building on these findings, *in vivo* experiments were undertaken using X-rays to develop a RE model in mice. Post-treatment with ACZF, marked improvements were noted in body weight and DAI scores in the RE mice. Histological evaluations of colon tissues indicated that ACZF treatment substantially reduced inflammatory cell infiltration and pathological alterations. Although colon Masson staining did not reveal significant differences between groups, this may be related to the early pathological characteristics of radiation-induced enteritis (primarily inflammation and epithelial damage) and the limitations of Masson staining (insensitivity to subtle collagen changes). Further WB analysis revealed increased levels of phosphorylated PI3K and AKT in the colons of the model group mice, indicating the activation of the PI3K/AKT pathway in the context of RE. The administration of ACZF led to a notable decrease in the phosphorylation levels of PI3K/AKT within the colon tissues of mice subjected to the RE model. Furthermore, simultaneous administration of ACZF alongside the PI3K activator YS-49 demonstrated that the inhibitory effects of ACZF on the PI3K/AKT pathway were not permanent, similarly affecting the expression levels of epithelial tight junction proteins and inflammatory mediators within the colonic mucosa. These findings reinforce the essential function of the PI3K/AKT pathway in the mitigation of RE by ACZF. Despite the lack of significant differences in Masson staining results, combined with other experimental findings, ACZF may exert its effects through anti-inflammatory, antioxidant, and intestinal barrier function improvement pathways. Future studies could employ more sensitive staining methods or multi-omics technologies to further explore the long-term effects of ACZF on collagen metabolism and its specific mechanisms of action.

Although this study combined with network pharmacology and animal experiments preliminarily revealed the potential mechanism of action of ACZF in the treatment of RE, there are still limitations. First of all, the mechanism exploration in this study focused on the overall activity changes of the PI3K/AKT pathway. However, the pathological mechanism of RE is complex, involving the interaction of multiple signaling pathways and molecular targets. Future studies could further explore upstream and downstream targets of the PI3K/AKT pathway (such as mTOR, GSK-3β, etc.), as well as other potentially relevant signaling pathways (such as NF-κB, MAPK, etc.). Secondly, because there is no standardized drug treatment regimen for RE, no positive control group was set up. Although this limited the direct comparison with other therapeutic drugs to a certain extent, this study fully verified the therapeutic effect of ACZF and its regulation of PI3K/AKT pathway by setting up a model group and a normal control group.

## 5 Conclusion

**FIGURE 7:**
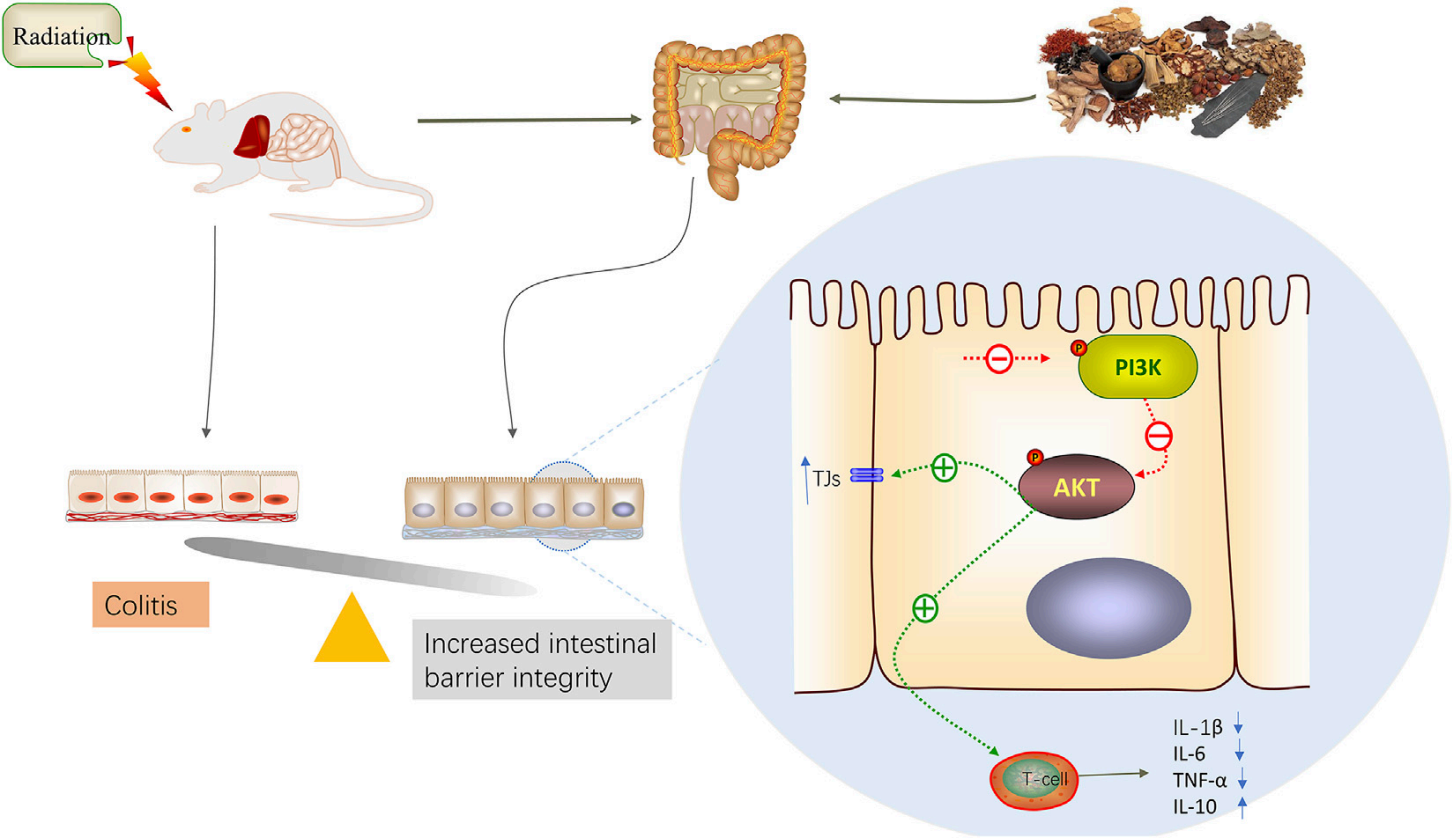
A model illustration of ACZF inhibits PI3K/AKT pathway, alleviates radiation-induced intestinal epithelial dysfunction and intestinal inflammation.

In summary, this study utilized network pharmacology to predict the components, targets, and mechanisms of action of the ACZF in treating RE. Through *in vivo* experiments, we confirmed the protective effects of the ACZF on the intestinal barrier function and its anti-inflammatory properties. Additionally, we observed the activation of the PI3K/AKT signaling pathway in radiation enteritis, with evidence that the ACZF can inhibit this pathway’s activation. Radiation exposure can lead to barrier damage and inflammation, and the protective effects of the ACZF against radiation enteritis are at least partially mediated by the inhibition of PI3K/AKT pathway activation (Figure 7).

## Data Availability

The datasets presented in this study can be found in online repositories. The names of the repository/repositories and accession number(s) can be found in the article/Supplementary Material.

## Supplementary material

SUPPLEMENTARY FIGURE S1 Flowchart of the preparation process of ACZF decoction.

SUPPLEMENTARY FIGURE S2 Total Ion Chromatogram (TIC) of compounds in ACZF decoction in positive ion mode **(a)** and negative ion mode **(b)**.

SUPPLEMENTARY TABLE S1 Different components in the formula of ACZF.

SUPPLEMENTARY TABLE S2 Table of information on secondary metabolites of ACZF detected by widely targeted metabolomics.
