# Supplementary figures and images for: Molecular mechanisms of the anchang group prescription in treating radiation enteritis: network pharmacology analysis and experimental evidence
Source: Front Pharmacol. 2025 Apr 8;16:1524925. doi: 10.3389/fphar.2025.1524925 (PMC12011760; doi:10.3389/fphar.2025.1524925)

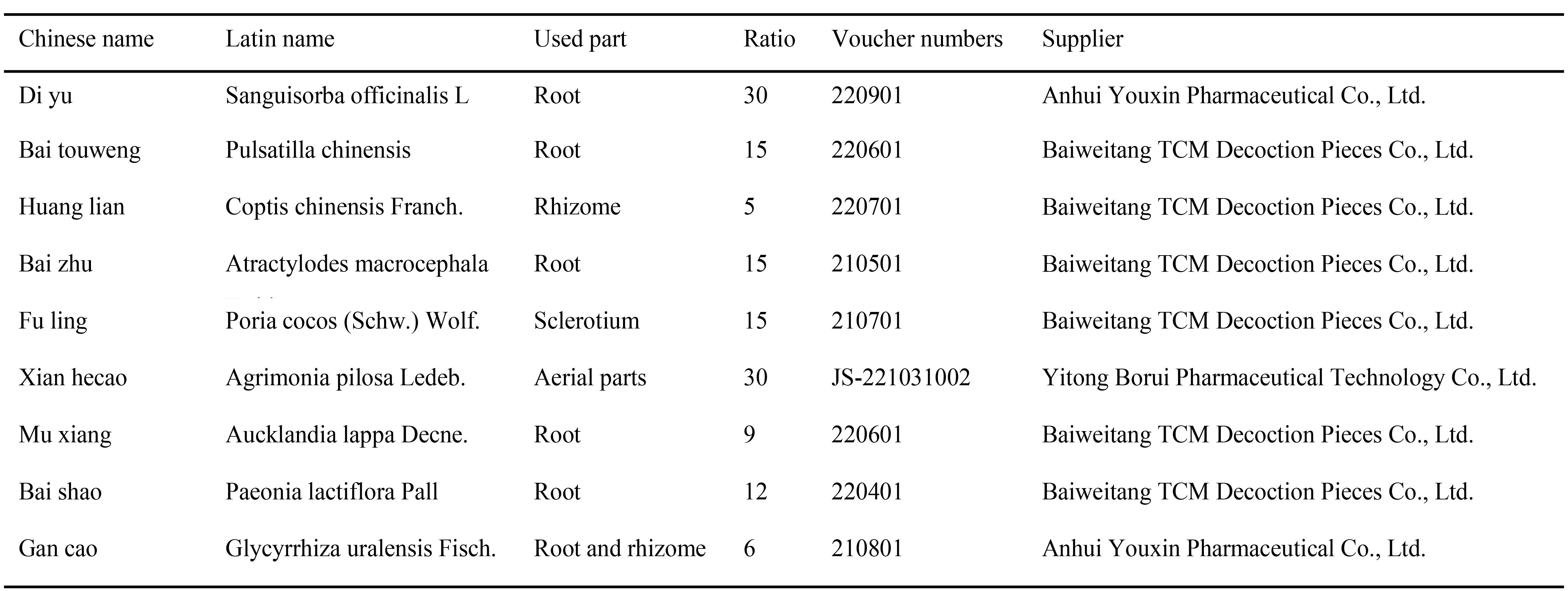

Supplement: Supplementary file 1 [file DataSheet1.zip › Table S2.TIF]

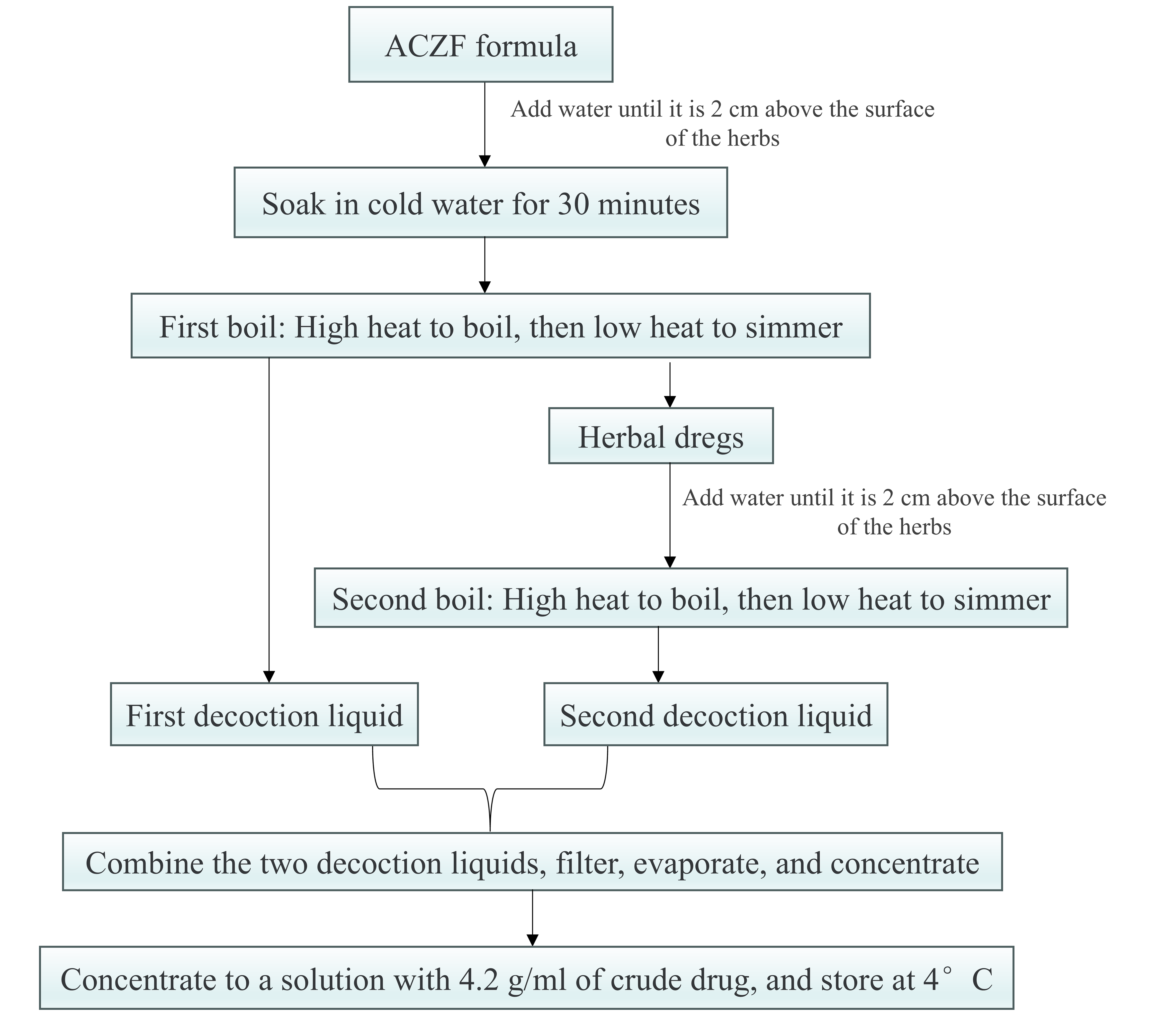

Supplement: Supplementary file 1 [file DataSheet1.zip › Figure S1.TIF]

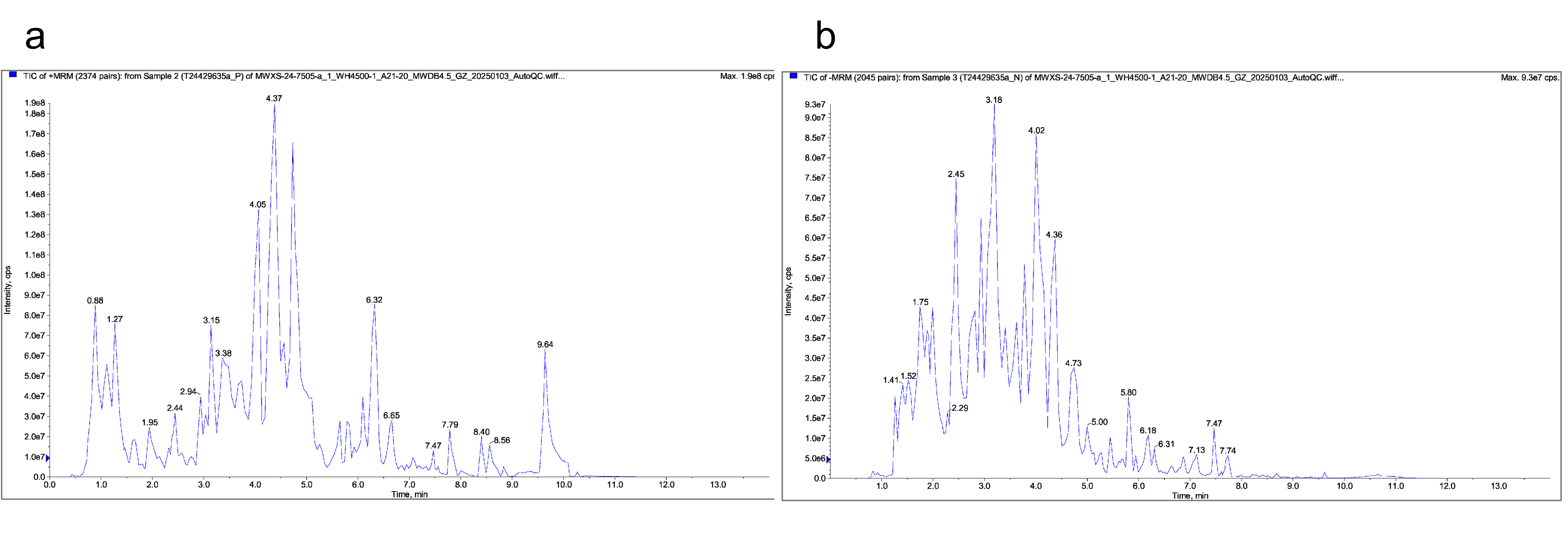

Supplement: Supplementary file 1 [file DataSheet1.zip › Figure S2.TIF]
